# Deep learning assisted sparse array ultrasound imaging

**DOI:** 10.1371/journal.pone.0293468

**Published:** 2023-10-30

**Authors:** Baiyan Qi, Xinyu Tian, Lei Fu, Yi Li, Kai San Chan, Chuxuan Ling, Wonjun Yim, Shiming Zhang, Jesse V. Jokerst

**Affiliations:** 1 Materials Science and Engineering Program, University of California San Diego, La Jolla, California, United States of America; 2 Department of Electrical and Electronic Engineering, The University of Hong Kong, Hong Kong SAR, China; 3 Department of NanoEngineering, University of California San Diego, La Jolla, California, United States of America; 4 Biomedical Engineering Program, The University of Hong Kong, Hong Kong SAR, China; 5 Department of Radiology, University of California San Diego, La Jolla, California, United States of America; Fudan University, CHINA

## Abstract

This study aims to restore grating lobe artifacts and improve the image resolution of sparse array ultrasonography via a deep learning predictive model. A deep learning assisted sparse array was developed using only 64 or 16 channels out of the 128 channels in which the pitch is two or eight times the original array. The deep learning assisted sparse array imaging system was demonstrated on *ex vivo* porcine teeth. 64- and 16-channel sparse array images were used as the input and corresponding 128-channel dense array images were used as the ground truth. The structural similarity index measure, mean squared error, and peak signal-to-noise ratio of predicted images improved significantly (p < 0.0001). The resolution of predicted images presented close values to ground truth images (0.18 mm and 0.15 mm versus 0.15 mm). The gingival thickness measurement showed a high level of agreement between the predicted sparse array images and the ground truth images, as indicated with a bias of -0.01 mm and 0.02 mm for the 64- and 16-channel predicted images, respectively, and a Pearson’s r = 0.99 (p < 0.0001) for both. The gingival thickness bias measured by deep learning assisted sparse array imaging and clinical probing needle was found to be <0.05 mm. Additionally, the deep learning model showed capability of generalization. To conclude, the deep learning assisted sparse array can reconstruct high-resolution ultrasound image using only 16 channels of 128 channels. The deep learning model performed generalization capability for the 64-channel array, while the 16-channel array generalization would require further optimization.

## Introduction

Two-dimensional (2D) and three-dimensional (3D) ultrasonography has been widely applied in imaging tissues and organs for diagnosing diseases due to its capability of serving as a real-time, non-invasive, portable, and radiation-free tool [1]. Ultrasound transducers are typically composed of multiple elements arranged in a linear or 2D array pattern for 2D and 3D imaging, respectively. Each element is controlled individually by an electrical channel, serving as a transmitter and receiver of ultrasound waves, which interfere with each other to create the ultrasound beam. At the same center frequency, image resolution is guided by the pitch, i.e., the spacing between individual elements in the ultrasound transducer array, and the number of transducer elements. The pitch is usually designed as one half of the ultrasonic wavelength in phased array ultrasound transducers [2], and close to one ultrasonic wavelength in linear array ultrasound transducers [3]. These dimensions are chosen to prevent imaging artifacts due to grating lobes, which are unwanted additional beams that can lead to spatial aliasing artifacts such as false echoes or image smearing. Such artifacts can compromise the image quality and accuracy of diagnostic information derived from the image [4]. Ultrasound transducers for 2D imaging usually have over one hundred transducer elements [5], and 2D array transducers for 3D imaging may have several thousands of elements [6]. The assembly of such dense transducers with so many elements is costly and complicated. In addition, the electrical control system to supply power and process signals for each channel becomes more complex and requires high power-consumption with more channels.

Sparse array ultrasound imaging has been reported to reduce the number and density of the transducer elements, including random array [7], non-uniform weighted array [8], circular ring array [9], non-grid optimal 2D array [10], etc. [11, 12]. However, these techniques suffer from low signal-to-noise ratio (SNR) due to lower ultrasound energy.

Deep learning methods have been applied in ultrasound imaging for automatic organ segmentation [13], object detection [14], as well as image resolution improvement [15]. For example, Nahas et al. have proposed a CNN-based deep learning approach to segment and resolve aliasing artifacts in ultrasound color flow imaging [16]. Liu et al. have built a self-supervised cycle generative adversarial network (GAN) to achieve perception consistency super-resolution ultrasound imaging. The cycle GAN only requires low-resolution ultrasound data to generate corresponding high-resolution images, and it ensures the re-degenerated images are consistent with the input low-resolution images, and vice versa [17]. More recently, Shin et al. have reported a super-resolution residual network based on a deep convolution to enhance the prediction quality of the transcranial focused ultrasound, providing potentials in minimizing the risk and improving the efficacy of non-invasive brain stimulation by acoustic pressure [18].

Deep learning technologies have also been proposed to improve the image quality of sparse array ultrasound imaging [4, 19]. The resolution and SNR of sparse array images via deep learning were comparable to traditional dense array imaging [20]. However, these existing works are only demonstrated on simulations or imaging phantoms with fixed structures rather than real tissue [21, 22]. Other works showed *in vivo/ex vivo* experiments were performed but with a pitch reduction of only two-fold (S1 Table) [4, 19, 23].

Here, a CNN-based robust UNet (RUNet) architecture was presented to restore the undersampled sparse array imaging with significant grating lobe artifacts [24]. The RUNet architecture was constructed by training image samples in a non-stationary way with a degradation model. Previous research has proved the RUNet effectiveness in generating high-resolution images with low reconstruction errors while possessing optimized visual quality [24]. Here, for the first time, the RUNet model was applied in biomedical ultrasonography.

In this study, the performance of the proposed CNN-assisted sparse array imaging was demonstrated on porcine teeth because of the value ultrasonography can have in oral health [25–27]. The porcine teeth were selected for three reasons: First, fresh porcine jaws could be easily obtained from a local abattoir. Second, the comparable size, enamel, and crown morphology make high similarity of porcine periodontal anatomy to that of humans [28]. Third, porcine teeth could be fixed in water for stable measurements using the ultrasound transducer to obtain images at the same spot.

Assisted with the RUNet model, a high-resolution sparse array imaging system has been demonstrated. Only 64 channels or 16 channels out of the total 128 channels were activated, and the pitch of the sparse array is eight or two times that of the original dense array. To the best of our knowledge, this investigation is the first to report on the use of an eight-fold pitch for sparse array ultrasound imaging, and it is also the first to apply the sparse array technique in periodontal applications.

## Methods and materials

### Study design

The CNN model was trained using 64- or 16-channel sparse array images as the input and 128-channel dense array images as the ground truth (i.e., reference). The performance of the CNN model was evaluated based on the improvement in image quality and the accuracy of landmarks localization accuracy of the predicted images. Please see the subsequent sections for more detailed information.

### Ultrasound imaging hardware system

Ultrasound transducer L35-16vX (Verasonics, Inc., Kirkland, USA) was used to collect ultrasound images of periodontal structures. The transducer has a linear array with 128 individual channels (28 MHz, -6 dB bandwidth >60%, 70 μm channel pitch, and 0.8 mm width along the elevation axis). By regularly turning off every other channel of the original 128-channel transducer, the 64-channel sparse array transducer has a pitch of 140 μm. Similarly, by regularly turning off seven out of eight channels of the original 128-channel transducer, the 16-channel sparse array transducer has a pitch of 560 μm. A 30-μm nichrome wire was immobilized in water under 10 mm from the surface of the transducer for characterizing the axial and lateral resolution of the B-mode imaging by the point spread function full width at half maximum (FWHM) (S1 Fig) [29]. The axial resolution of 128-, 64- and 16-channel imaging is 470 μm, 430 μm and 1750 μm, respectively. The lateral resolution of 128-, 64- and 16-channel imaging is 320 μm, 320 μm and 2550 μm, respectively.

A data acquisition (DAQ) system (Vantage 256, Verasonics, Inc., Kirkland, USA) was connected to the transducer to provide a 15-V power supply, generate pulses with a repetition rate of 5 kHz, sample the radiofrequency data of each channel, and reconstruct ultrasound images (S2 and S3 Figs). The DAQ has a frequency range of 2 to 42 MHz, 14-bit A/D converters with a programmable sample rate up to 62.5 MHz, and can image up to 100,000 frames/second.

### Ultrasound image reconstruction

B-mode ultrasound imaging with a size of 512 pixels × 512 pixels was performed with coherent compounding beamforming by applying a series of time-delayed electrical excitation in each transducer channels [30]. Hanning window apodization was applied as a weighting function across the transducer aperture [31]. Plane ultrasonic waves at seven different angles evenly distributed from -15° to 15° were generated in a row to scan the field of ultrasound imaging from different orientations [32]. By averaging the reflected ultrasound signals at all seven tilting angles, one image frame was reconstructed.

### Preparation and imaging of porcine teeth

**Fig 1:**
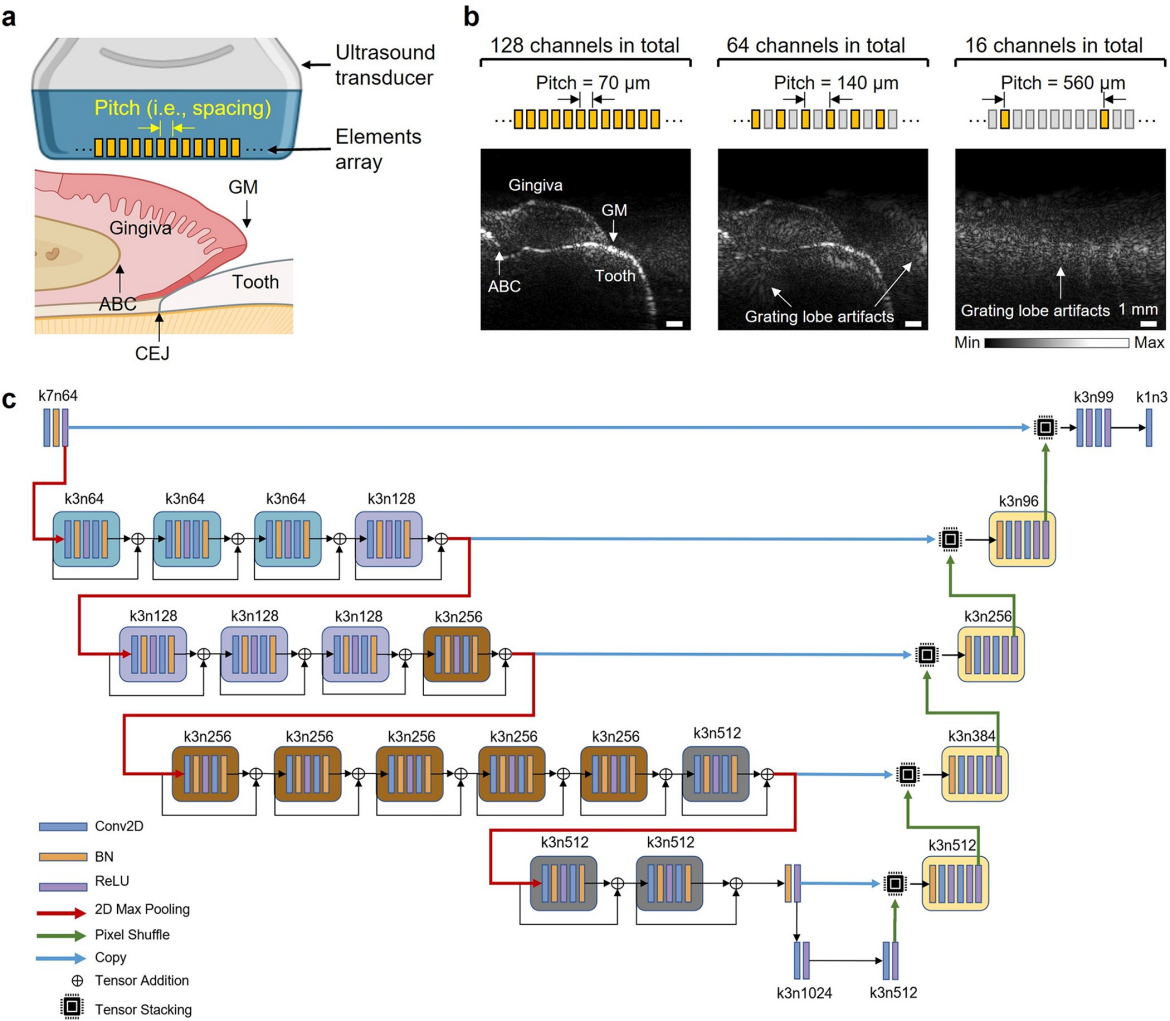
The overview of deep learning-assisted sparse array periodontal imaging. (a) Schematics of imaging periodontal anatomy using an ultrasound transducer. The ultrasound transducer includes an array of elements of a specific pitch (i.e., spacing). Periodontal structural anatomy includes the tooth, gingiva, alveolar bone crest (ABC), cementoenamel junction (CEJ), and gingival margin (GM). (b) Periodontal imaging reconstructed from 128-, 64- and 16-channel ultrasound transducer. By regularly turning off every other channel of the original 128-channel transducer, the pitch of 64-channel is 140 μm. Similarly, by regularly turning off seven out of eight channels of the original 128-channel transducer, the pitch of 16-channel is 560 μm. (c) The CNN-based RUNet architecture that predicts high-quality images from the sparse array imaging input. Conv2D: convolutional layer. BN: batch norms. ReLU: rectified linear units.

Fresh porcine jaws were purchased from a local abattoir and prepared as previously reported [33]. The porcine head was sagittally sliced, and the mandible and maxilla were separated. The porcine jaw, which includes teeth and periodontal tissues, was immersed in water for ultrasound imaging coupling. Ultrasound provides visualization of tooth anatomical features (Fig 1A) including alveolar bone crest (ABC), cementoenamel junction (CEJ), gingival margin (GM), etc. Fig 1B compares reconstructed images with the original 128-channel dense array and the 64- or 16-channel sparse array in a representative tooth.

### Architecture and training of the RUNet model

The RUNet is composed of two sections of dimensionally symmetric convolutional layers: encoder and decoder (Fig 1C). The encoder section analyzes and extracts features of the input images. In between the convolutional layers, a pooling layer downsamples the images and contracts the image size by half. The decoder section generates results based on the extracted features from the encoder section. The images are expanded to upsample the images until the dimension reached the same as the images input to the first layer of encoder. The RUNet consists of a series of convolutional layers (Conv2D), batch norms (BN), ReLU activation functions, and tensor addition operations [24]. The tensor addition operation feeds forward the same block input to the subsequent block. Additionally, the sub-pixel convolutional layers in the expansive path for feature expansion are utilized to upscale the resolution of images. On top of that, perceptual loss functions are also introduced to characterize the perceptually relevant features between inputs and predictions. A python-based RUNet programme was designed to establish a pipeline for high-resolution sparse array ultrasound image reconstruction. The programme first shuffled and preprocessed the ultrasound images to build the training dataset, validation dataset and test dataset. Then the RUNet model was trained with the datasets and the training process was operated on a Tesla V100 GPU (provided by Google Colaboratory). After the validation loss decreased to an acceptable value, the current model parameters were frozen and output into an h5 file. After the training process, the model was rebuilt with the best parameters and the test dataset was input into the model to validate the model performance.

### Preparation of datasets

**Fig 2:**
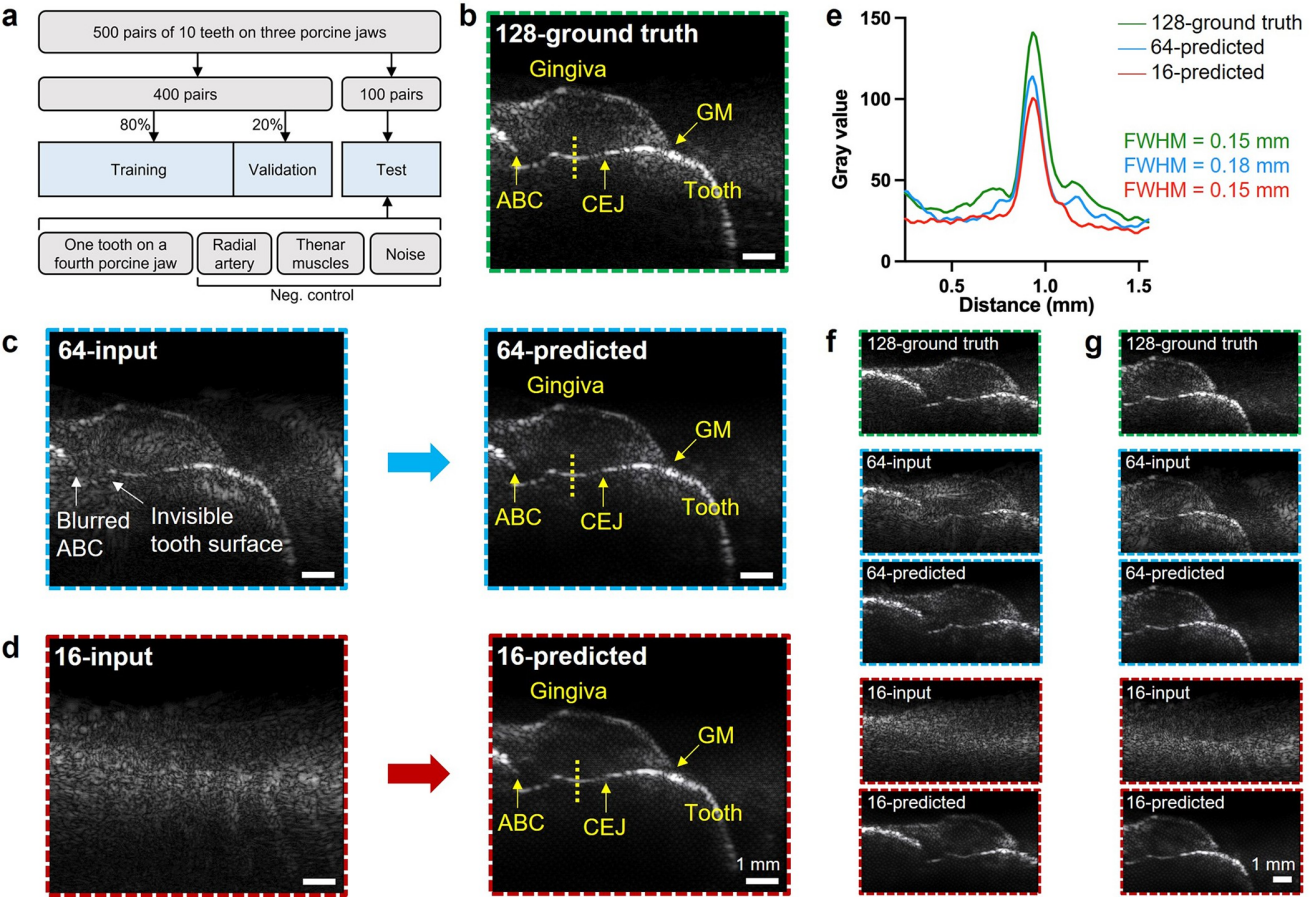
CNN prediction of a representative tooth (4^th^ pre-molar). (a) Datasets of deep learning model training, validation, and test. (b) The ground truth image reconstructed from the original 128-channel dense array. (c) Undersampled 64-channel imaging (left) versus its artifact-free output (right) predicted by the CNN model. (d) Undersampled 16-channel imaging (left) versus its artifact-free output (right) predicted by the CNN model. (e) One-dimensional line spread function profiles across the reconstructed tooth surface extracted from 128-channel ground truth, 64-channel predicted, and 16-channel predicted image. FWHM: full width at half maximum. Ground truth, input, and predicted images of the representative tooth at posterior position (f) and anterior position (g).

A collection of 1500 cross-sectional images with a size of 512 × 512 pixels from 10 teeth of three porcine jaws was obtained using three different transducer channel configurations (128, 64, and 16 channels). At the same position of the same tooth, one image was captured using each of the three transducer channel configurations. As a result, the image dataset consists of 500 image pairs each pair containing one 128-channel image, one 64-channel image, and one 16-channel. Of the 500 image pairs, 400 pairs were divided into training (80%) and validation (20%) datasets. The images captured with the 64- and 16-channel transducers were labeled as sparse array inputs, while the images captured with the 128-channel transducer were labeled as ground truth. The remaining 100 image pairs served as the test dataset. The test dataset also contained an image of a tooth from a previously unseen fourth porcine jaw, images of the radial artery and thenar muscles, and images of noises. The inclusion of a tooth image from a new porcine jaw could evaluate the generalization capability of the predictive model. The images of radial artery, thenar muscles, and noises were collected as negative control to investigate the over-fitting possibility of the deep learning model (Fig 2A).

The images were manually analyzed and measured. Before collecting the 64- and 16-channel sparse array images, the examiner had to ensure that all 128-channel ground truth images met specific quality criteria, which included: (1) identifying the GM and gingival surface, (2) identifying the tooth surface, and (3) ensuring there were no interfering artifacts coincident with the relevant anatomy. If these conditions were met, further image collection was performed.

### Characterization of image quality improvement

The original images were compared to the reconstructed images. This comparison used established metrics known as the structural similarity index measure (SSIM), mean squared error (MSE), and peak signal-to-noise ratio (PSNR). These metrics were measured in MATLAB to quantitatively compare the image quality of the input sparse array images with the images predicted by the CNN model. SSIM was used to measure the structural similarity between two images [34]. SSIM ranges from -1 to 1, with a value of 1 indicating a perfect match, 0 indicating no similarity, and -1 indicating complete dissimilarity. SSIM values closer to 1 suggest a greater degree of similarity between two images.


$$SSIM=\frac{\left(2\mu_{GT}\mu_{CNN}+k_{1})({2\sigma }_{cov}+k_{2}\right)}{\left(\mu_{GT}^{2}+\mu_{CNN}^{2}+k_{1})(\sigma_{GT}^{2}+\sigma_{CNN}^{2}+k_{2}\right)}$$

Here, μ_GT_ (σ_GT_) and μ_CNN_ (σ_CNN_) represent the pixel sample mean (variance) of the ground truth images and CNN input/predicted images, respectively. σ_cov_ is the covariance of ground truth images and CNN input/predicted images. k_1_ and k_2_ are two variables to stabilize the division with a weak denominator.

MSE calculated the corresponding pixel value differences of two images [34]. MSE ranges from 0 to a positive value, with 0 indicating a perfect match. MSE values closer to 0 suggest a higher similarity between two images.


$$MSE=\frac{1}{MN}\sum_{m=0}^{M-1}\sum_{n=0}^{N-1}{\left(I_{GT}(m,n)-I_{CNN}(m,n)\right)}^{2}$$

Here, I_GT_ and I_CNN_ are the ground truth and CNN input/predicted images of sizes of M×N, respectively.

PSNR measures image quality by comparing the maximum possible power of the original image to the power of the noise introduced during compression or processing [35]. Higher PSNR values indicate better image quality as they reflect less distortion.


$$PSNR=20{log}_{10}(\frac{I_{max}}{\sqrt{MSE}})$$

Here, I_max_ indicates the maximum possible values in the given images.

### Intersection over union (IoU) index

The gingiva region was manually segmented with ImageJ by drawing the boundary along the gingiva surface, the GM, and then the tooth surface on a 28-inch monitor with resolution of 3840 pixels × 2160 pixels [36]. The IoU index was calculated using MATLAB to measure the similarity between the gingiva region in 64- and 16-channel predicted images and 128-channel ground truth images. IoU is defined as the size of intersection divided by the union of the region in two images [37].

### Measurement of gingival thickness

Gingival thickness was measured using ImageJ software from the reconstructed ultrasound images [38]. For consistency, all measurements were made 2 mm from the GM. A perpendicular line was drawn from the tooth surface to the surface of the gingiva. The length of this line was recorded as the gingival thickness with a precision of 0.01 mm (S4 Fig). Gingival thickness measurements were performed on the 128-ground truth, 64-predicted, and 16-predicted images. The measurement of all ten porcine teeth was performed in duplicate by two blinded examiners and averaged. Examiner 1 (B.Q.) was an ultrasound researcher with four years of ultrasound experience. Examiner 2 (C.L.) had no prior experience with ultrasound and received brief training to identify the landmarks in ultrasound images for gingival thickness measurements. On three representative teeth with large (Tooth A), medium (Tooth B), and small (Tooth C) gingival thicknesses, both examiners performed three replicates of gingival thickness measurements to study the reproducibility.

In the clinical transgingival probing method, a 28-gauge needle with calipers was inserted into the gingiva perpendicularly through the gingival surface until the needle touched the tooth surface [39]. By marking a line on the needle, the gingival thickness was obtained by measuring the distance between the marked line and the needle tip with a 0.1 mm precision caliper. The clinical probing method was performed by Examiner 1.

### Statistical analysis

A power analysis was conducted using G*Power to estimate the adequacy of the sample size (n) for determining differences in gingival thickness measurements between images grouped as 128-ground truth vs. 64-predicted and 128-ground truth vs. 16-predicted, respectively, using a two-tailed significance test with a 95% significance (α = 0.05), and minimum differences of 0.3 mm, 0.5 mm, or 1.0 mm [40]. Each of the two comparison groups consisted of ten teeth, resulting in a total sample size of n = 20 included in this study. The power-sample size relationship was computed over a range of n = 4 to 60.

Bland-Altman analysis was performed to characterize the bias and limits of agreement between measurements of grouped predicted images and ground truth images, and between two image examiners. Pearson correlation was performed to quantify the agreement between grouped images. A paired t-test was applied to justify if the measurements of grouped images were significantly different. A p-value < 0.0001 was considered significant. Bland-Altman analysis, Pearson correlation, and paired t-test were performed using GraphPad Prism version 9.5.0 (San Diego, CA, USA).

## Results

### Artifact correction from undersampled sparse array images

A representative image of porcine tooth (4^th^ pre-molar) reconstructed from the 128-channel transducer was shown in Fig 2B. Notably, while the anatomy remains visible in the 64-channel image (Fig 2C, left), the grating lobe artifacts reduce the visibility of the anatomical features. For example, the ABC cannot be distinguished from the background, and the tooth surface below the CEJ is merged with the artifacts. There are no detectable anatomical features in the 16-channel image (Fig 2D, left). The results predicted from 64- and 16-channel were presented in Fig 2C and 2D, right, respectively. Fig 2E showed the yellow dashed line profile crossing the tooth surface of the 128-channel ground truth, 64-channel predicted, and 16-channel predicted images. After image processing by the CNN-model, the FWHM of 64- and 16-channel predicted images is 0.18 mm and 0.15 mm, respectively. The resolution of CNN-assisted sparse array imaging is comparable to the dense array imaging ground truth (0.15 mm). On the same tooth, the CNN-assisted sparse array imaging at different positions were shown in Fig 2F and 2G.

### SSIM, MSE, and PSNR improvement of predicted images

**Fig 3:**
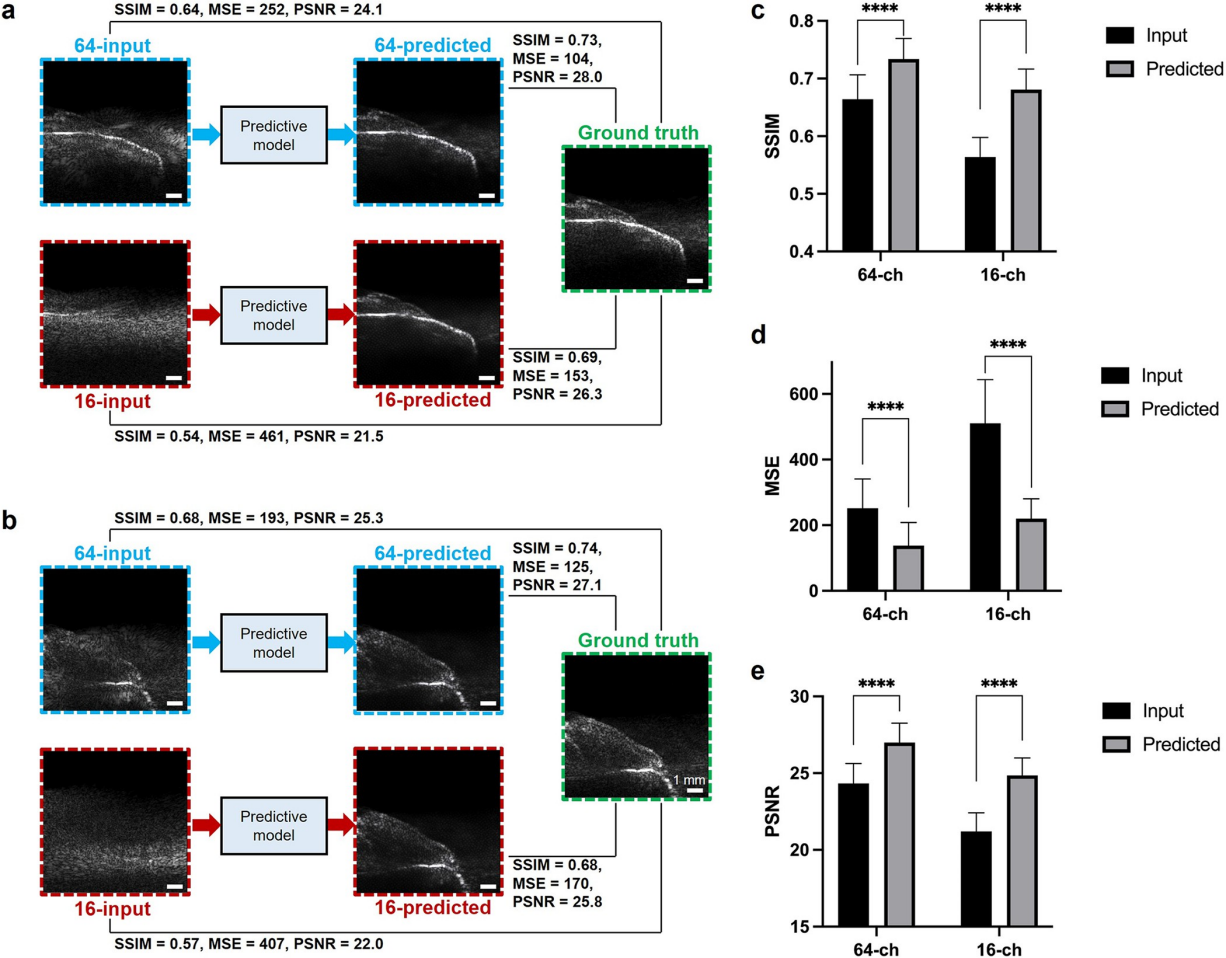
Characterization of the CNN-predicted image quality improvement. Input and predicted images by 64- and 16-channel sparse array, and the ground truth images by the original 128-channel dense array of two representative teeth: (a) 2nd pre-molar and (b) 3rd molar. (c) SSIM, (d) MSE, and (e) PSNR of the 128-channel ground truth image versus 16- and 64-channel input and predicted images of all 100 pairs of images in the entire test dataset. ****: p < 0.0001. SSIM: structural similarity index measure. MSE: mean squared error. PSNR: peak signal-to-noise ratio.

Input, predicted, and ground truth images of two representative porcine teeth are shown in Fig 3A and 3B. After being restored by the predictive model, 100 pairs of images in the entire test dataset indicated that the mean SSIM significantly improved from 0.66 to 0.73 in the 64-channel sparse array imaging, and from 0.56 to 0.68 in the 16-channel sparse array imaging (p < 0.0001) (Fig 3C). The MSE significantly improved from 252 to 137 in the 64-channel imaging, and from 511 to 220 in the 16-channel imaging (p < 0.0001) (Fig 3D). The PSNR significantly improved from 24.3 to 27.0 in the 64-channel sparse array imaging, and from 21.2 to 24.9 in the 16-channel sparse array imaging (p < 0.0001) (Fig 3E). The improvement of SSIM, MSE, and PSNR is summarized in S2 Table.

The improvement of CNN model prediction accuracy with increasing numbers of epochs was characterized next (S5 Fig). The shape of the tooth anatomy was predicted at 50 epochs. This did not improve when the number of epochs was further increased to 100. Thus, 50 epochs were used to train the CNN model in the following experiments.

### Gingival thickness measurement

**Fig 4:**
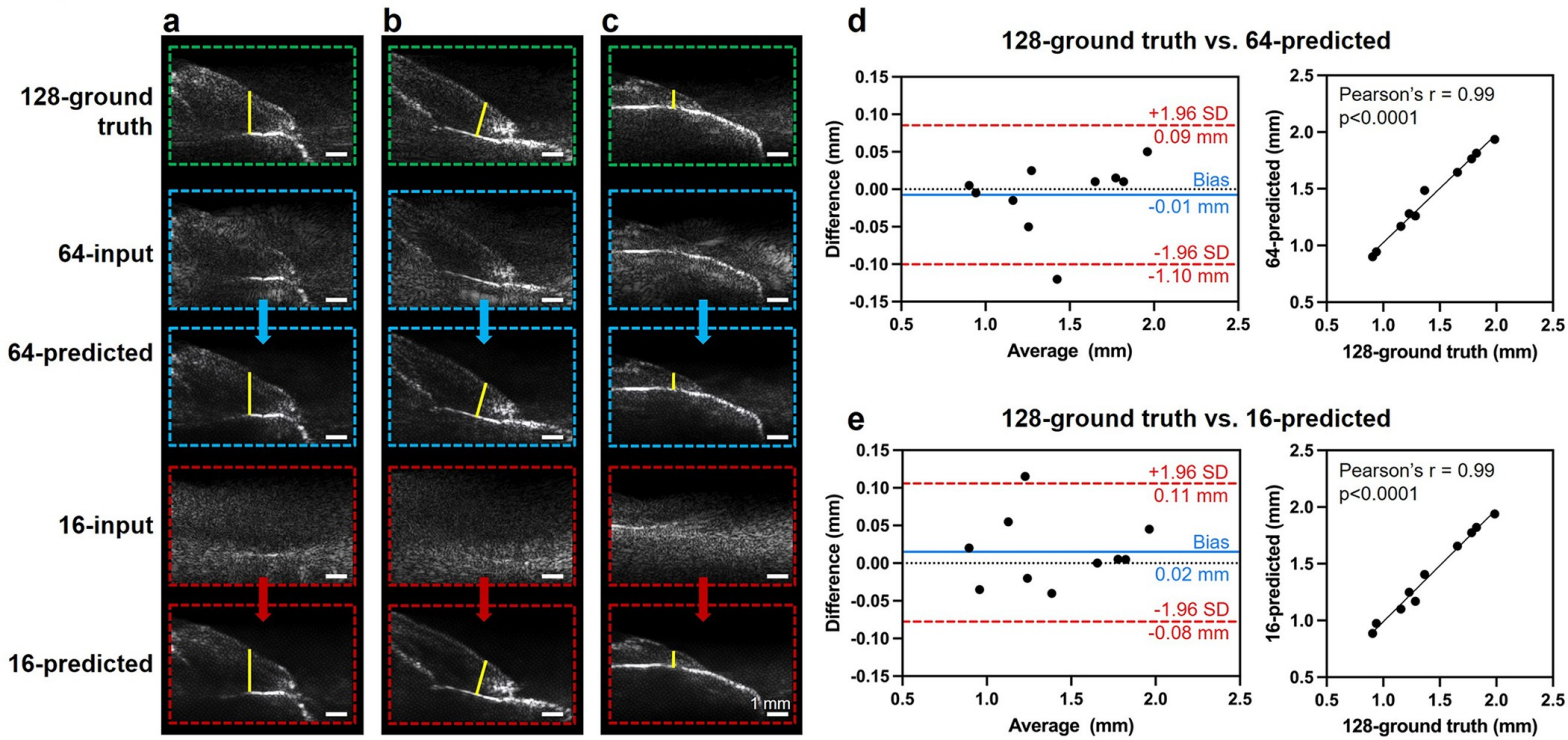
Gingival thickness measurement comparison between ground truth and predicted images. Gingival thickness (marked as the yellow line) measured from 128-channel ground truth image, 64-channel predicted image, and 16-channel predicted image on three representative teeth with (a) large, (b) medium, and (c) small gingival thickness. Bland-Altman plot and Pearson correlation of gingival thickness measurement comparison of (d) 128-ground truth vs. 64-predicted and (e) 128-ground truth vs. 16-predicted.

The gingival thickness of ten different teeth with varying thicknesses was measured in the paired 128-ground truth, 64-predicted, and 16-predicted images (S6 and S7 Figs), and summarized the descriptive statistics in Table 1. Images of three representative teeth with large (Fig 4A), medium (Fig 4B), and small (Fig 4C) gingival thicknesses were presented here. The gingival thickness measurement showed a high level of agreement in the ground truth images and the predicted images, as indicated by the Bland-Altman analysis with a bias of -0.01 mm and the Pearson’s r = 0.99 (p < 0.0001) for the comparison between 128-ground truth vs. 64-predicted images (Fig 4D). Similarly, the bias was 0.02 mm and the Pearson’s r = 0.99 (p < 0.0001) when comparing 128-ground truth vs. 16-predicted images (Fig 4E). The p-values were 0.63 for 128-ground truth vs. 64-predicted and 0.34 for 128-ground truth vs. 16-predicted, respectively, thus indicating no significant difference. The threshold for significance was set as p < 0.0001. The means, standard deviations, and relative standard deviations of three replicates on three representative teeth A, B, and C were summarized in S3 Table for Examiner 1, and S4 Table for Examiner 2. The relative standard deviations were up to 4%. For all ten teeth, the gingival thickness measurement bias between the two examiners was -0.03 mm in 128-ground truth images, -0.04 mm in 64-predicted images, and -0.04 mm in 16-predicted images (S8 Fig).

**Table 1:** Descriptive statistics of all gingival thickness measurements (in mm).

Methods	N	Mean (SD)	Min	Max
**128-ground truth**	10	1.41 (0.38)	0.91	1.99
**64-predicted**	10	1.42 (0.37)	0.90	1.94
**16-predicted**	10	1.40 (0.38)	0.89	1.94

SD: standard deviation.

The power analysis indicated that the sample size of gingival thickness measurement comparison in this study (n = 20) had sufficiently high power (≥ 80% by convention) to detect a minimum difference of ≥ 0.5 mm (S9 Fig). Finally, the ultrasound imaging-based gingival thickness measurement was compared to the clinical transgingival probing on three representative teeth (S5 Table) [39]. The mean differences were 0.01 mm, 0.03 mm, and 0.03 mm for clinical probing vs. 128-ground truth, clinical probing vs. 64-predicted, and clinical probing vs. 16-predicted, respectively.

### Network generalization

**Fig 5:**
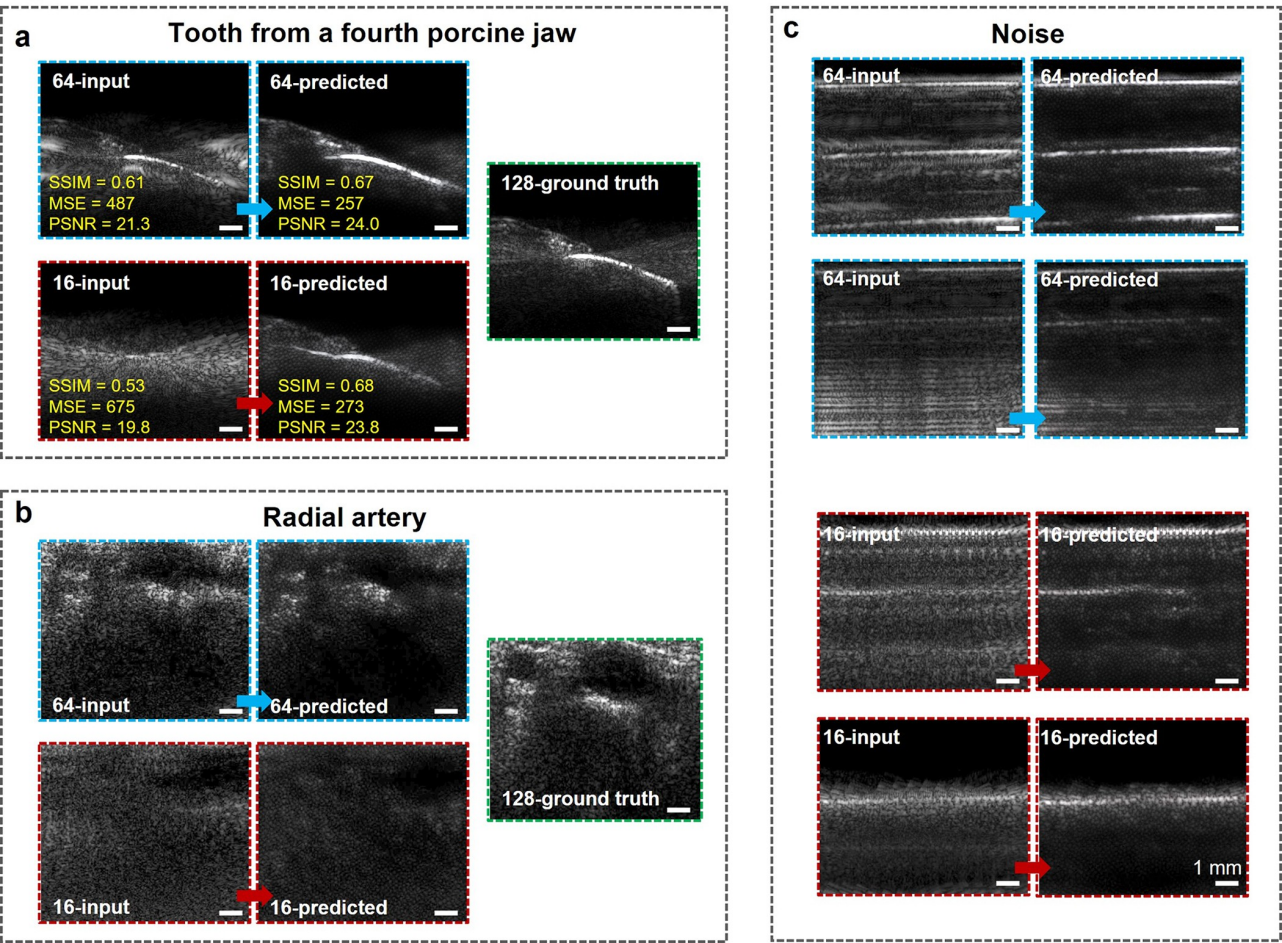
Investigation on the generalization capability and over-fitting possibility of the CNN model. Input and predicted images by 64- and 16-channel sparse array, and the ground truth images by the original 128-channel dense array of (a) a tooth from a new porcine jaw that was separate from the training dataset, and (b) radial artery. (c) Input and predicted images by 64- and 16-channel sparse array of noise signals.

Next, as a test of practical utility and generalization, the deep learning algorithm was used to image a porcine tooth (1st molar) completely separate from the training dataset (Fig 5A). The visibility of anatomical structures (e.g., gingiva, tooth surface, and GM) increases in both 16- and 64-channel predicted images. For the 64-channel images, SSIM improved from 0.61 to 0.67, MSE improved from 487 to 257, and PSNR improved from 21.3 to 24.0. For the 16-channel images, SSIM improved from 0.53 to 0.68 (S10 Fig), MSE improved from 675 to 273, and PSNR improved from 19.8 to 23.8. For the tooth from a separate porcine jaw, the IoU of gingiva region was 0.97 between 64-predicted vs. 128-ground truth, and 0.73 between 16-predicted vs. 128-ground truth. As comparison, for a tooth that was included in the training dataset, the IoU of the gingiva region 0.96 for both 128-ground truth vs. 64-predicted and 128-ground truth vs. 16-predicted (S11 Fig).

### Investigating network over-fitting possibility

Finally, a group of ultrasound images not from tooth were input as the test dataset to serve as negative control, including images on radial artery (Fig 5B), noise (Fig 5C), and thenar muscles (S12 Fig). The predicted images did not contain any features of tooth anatomy landmarks, which addressed that our CNN model does not have over-fitting issue.

## Discussion

According to the Huygens principle, the pitch of linear array ultrasound transducer is required to be close to one wavelength to form a single wave front from wave fronts from each element [3]. When the pitch is much larger than the wavelength, the interference of ultrasound waves from each element would generate unwanted grating lobes and degrade the image quality, which is the limitation of sparse array imaging [4]. In this study, a predictive CNN model was introduced to assist in restoring the sparse array imaging and improving the image resolution. Benefiting from the powerful information analysis and optimization capability, the typical UNet architecture has demonstrated effective results for improving image quality when trained with limited data [41]. However, when handling the sparse array US imaging, the non-stationary degradations of images can result in unstable training and underperform of the transitional UNet methods. Motivated by the residual neural network, Hu et al. reported a robust UNet (RUNet) that can learn more complex structures compared with the conventional UNet and restore degraded low-resolution images for image super-resolution [24]. Distinguished from the conventional UNet model, newly-designed residual blocks consist of Conv2D, BN, ReLU activation functions, and a followed tensor addition operation. The tensor addition operation enhances the ability of the model to learn complicated samples. The sub-pixel convolutional layers are utilized to upscale the resolution of images efficiently. All of these performance enhancement schemes result in significant gains in perceptual quality when the RUNet model is handling complicated and low-resolution images. Here, the RUNet model was firstly applied in biomedical ultrasonography.

In the 64-channel sparse array imaging, the grating lobe artifacts reduce the resolution, which leads to inaccurate anatomical feature localization. The ABC cannot be distinguished from the background, and the tooth surface below the CEJ is merged with the artifacts. There are no detectable anatomical features in the 16-channel input image due to the huge pitch that was of ten times of the transducer wavelength. The RUNet predictive model can eliminate artifacts and differentiate the anatomical features from the background. The quality of the 64- and 16-channel output images predicted by the algorithm is comparable to the ground truth images reconstructed with the 128-channel transducer as indicated by the close resolution of tooth surface (0.18 mm for the 64-channel and 0.15 mm for the 16-channel vs. 0.15 mm for the 128-channel). The SSIM, MSE, and PSNR significantly improved (p < 0.0001) when comparing the predicted images to the input sparse array images, showing the considerable image quality improvement by the CNN model.

Additionally, the accuracy of morphology in the predicted images was characterized by measuring a clinical periodontal index: the gingival thickness. Based on an ex vivo study conducted on 20 porcine cadavers, Kloukos et al. reported that clinical transgingival probing can be considered as reliable for assessing gingival thickness values [42]. Comparing to the invasive clinical probing, ultrasound has been reported as a non-invasive alternative tool for gingival thickness measurement. In this study, gingival thickness results measured from 64- and 16-channel predicted images showed high agreement and no significant difference with the 128-ground truth images. The bias for gingival thickness measured with predicted sparse array ultrasound images versus clinical probing needle was negligible (< 0.05 mm), which is less than the precision of the clinical probing method (0.1 mm). The proposed sparse array technique is highly reproducible with relative standard deviations of three replicates up to 4% and inter-examiner bias < 0.05 mm. Clinically, gingival thickness is assessed in a binary manner by evaluating the probe visibility after inserting the periodontal probe into the gingival sulcus [43]. The gingival biotype is classified as thin if the probe is visible due to transparency and thick if the probe is not visible due to thickness. A value of <1.0 mm and >1.0 mm is proposed for thin and thick biotype, respectively [44]. In comparison, ultrasound imaging provides a high-resolution tool (0.01-mm precision) for quantitative measurement of gingiva thickness. These results indicated that our CNN-assisted sparse array imaging can provide accurate anatomical landmarks localization and have potential value to periodontology.

In the generalization test using a separate porcine tooth as the input, the reconstructed shape of anatomical structures in the 64-channel predicted image are consistent with that of the 128-channel ground truth image, as indicated by the high IoU of the gingiva region (0.97). The generalization capability of 64-channel imaging provides potential for using the *ex vivo* porcine teeth as a training dataset to train the CNN model. One can then possibly apply the well-trained model to human subjects. In this way, redundant efforts in collecting hundreds of datasets in human subjects could be avoided.

The 16-channel predicted image could reproduce the anatomy of the gingiva and tooth in a generalization test but with reflection signals below the GM. These reflections are not seen in the 128-channel ground truth images. Also, the gingival surface morphology is not consistent with the ground truth image. The 16-channel input sparse array images are more undersampled than the 64-channel. Thus, it is more challenging for the CNN model to generalize the 16-channel prediction effectively. Clinically, a 16-channel sparse array is probably unlikely to be needed. Transfer learning could be applied to the unseen new subjects and/or further enlarge the training dataset to improve the generalization of 16-channel sparse array.

The RUNet CNN model applied in this study was trained by supervised learning, which required paired low-resolution and high-resolution images for training. However, obtaining paired data could be challenging and impractical in clinical settings. In 2017, Ledig et al. reported a super-resolution GAN model that could generate photo-realistic super-resolution results [45]. The GAN was trained using an unsupervised approach, which is applicable in biomedical imaging field when paired data are not available. GAN-based deep learning method has also been proposed to improve image resolution of ultrasound images [17]. Nevertheless, the training of GAN suffers from computational complexity, generating data with little diversity, mode collapse, non-convergence and instability [46]. Thus, it requires optimal hyperparameter tuning and long training times.

## Conclusions

This study reports a deep learning-assisted sparse array ultrasound imaging system to reduce the cost of fabrication, complexity of channel control, and electrical power consumption. The proposed sparse array system only requires 1/8 of the traditional ultrasound transducer channels to generate high-resolution images that have comparable quality of the original dense array.

The SSIM enhances from 0.66 to 0.73 when using 64 out of 128 channels and from 0.56 to 0.68 with using only 16 out of 128 channels. The resolution of the sparse array image predicted by the CNN model is close to that of the original dense array (0.18 mm for the 64-channel and 0.15 mm for the 16-channel versus 0.15 mm for the 128-channel). The deep learning model shows promising generalization capability for use in more subjects/sites. Negative controls proved that the model did not over fit during the training process. Future work will include improvements in device instrumentation (e.g., reducing the number of active transducer elements and demonstrating the sparse array imaging system in a 2D array for 3D volume imaging). Additional optimization of increasing the generalization capability will include enlarging the datasets to more diverse imaging targets and introducing transfer learning in the deep learning algorithms. Besides imaging the tooth anatomy, the sparse array imaging application could be further expanded in imaging different tissues and organs (e.g., heart, liver, kidney, etc.) to evaluate the cardiovascular diseases. Clinically, the sparse array imaging could be transferred from porcine teeth to human subjects (healthy and diseased) for clinical translation.

## Supporting information

S1 Fig Characterization of imaging resolution of 128-, 64- and 16-channel ultrasound probe. (a) Wire imaging reconstructed from 128-, 64- and 16-channel ultrasound probe. (b) Characterization of axial (left) and lateral (right) resolution of the 128-, 64- and 16-channel probe.
(TIF)

S2 Fig Electrical controlled delay-and-sum beamforming of each channel in the transducer array for signal processing [47]. (TIF)

S3 Fig Schematics of the ultrasound imaging system. The system includes ultrasound transducer and DAQ system. The ultrasound transducer is connected to DAQ system for power supply, data processing and real-time imaging display.
(TIF)

S4 Fig The gingival thickness measurement using ultrasound imaging method. Red line: 2-mm from the GM. Yellow line: gingival thickness. GM: gingival margin.
(TIF)

S5 Fig Improvement of predicted image quality with increasing number of epochs. (TIF)

S6 Fig Ten different teeth for gingival thickness measurement in 128-ground truth and 64-predicted images. The scale bar applies to all images.
(TIF)

S7 Fig Ten different teeth for gingival thickness measurement in 128-ground truth and 16-predicted images. The scale bar applies to all images.
(TIF)

S8 Fig Inter-examiner bias and agreement using Bland-Altman analysis. (a) 128-ground truth, (b) 64-predicted, and (c) 16-predicted images.
(TIF)

S9 Fig Statistical power as a function of sample size with three effect sizes calculated using two-tailed significance test with a 95% significance (α = 0.05). Mean differences were 0.3 mm (red), 0.5 mm (blue), and 1.0 mm (green). Effect size was calculated using gingival thickness measurement standard deviations of (a) 128-ground truth vs. 64-predicted and (b) 128-ground truth vs. 16-predicted, respectively.
(TIF)

S10 Fig Local SSIM map of the separate porcine tooth. SSIM: structural similarity index measure.
(TIF)

S11 Fig IoU of the gingiva region. (a) In the new tooth from a new porcine jaw, the IoU of gingiva region was 0.97 for 128-ground truth vs. 64-predicted and 0.73 for 128-ground truth vs. 16-predicted. (b) In one of the ten teeth in the training dataset, the IoU of gingiva region was 0.96 for 128-ground truth vs. 64-predicted and 0.96 for 128-ground truth vs. 16-predicted. Yellow, blue, and red regions indicate the gingiva region of 128-ground truth, 64-predicted, and 16-predicted image, respectively. Green and orange regions indicate the intersection of the 128-ground truth with the 64-predicted and 16-predicted, respectively. IoU: intersection over union.
(TIF)

S12 Fig Input, predicted, and ground truth images of thenar muscles. (TIF)

S1 Table Comparison between our work and published deep learning-assisted sparse array imaging papers. (DOCX)

S2 Table Summary of SSIM, MSE, and PSNR improvement. (DOCX)

S3 Table Summary of three gingival thickness replicates by Examiner 1 (B.Q.) (in mm). (DOCX)

S4 Table Summary of three gingival thickness replicates by Examiner 2 (C.L.) (in mm). (DOCX)

S5 Table Comparison between ultrasound and clinical probing of three gingival thickness measurements (in mm). (DOCX)
