# Supplementary figures and images for: Deep learning assisted sparse array ultrasound imaging
Source: PLoS One. 2023 Oct 30;18(10):e0293468. doi: 10.1371/journal.pone.0293468 (PMC10615290; doi:10.1371/journal.pone.0293468)

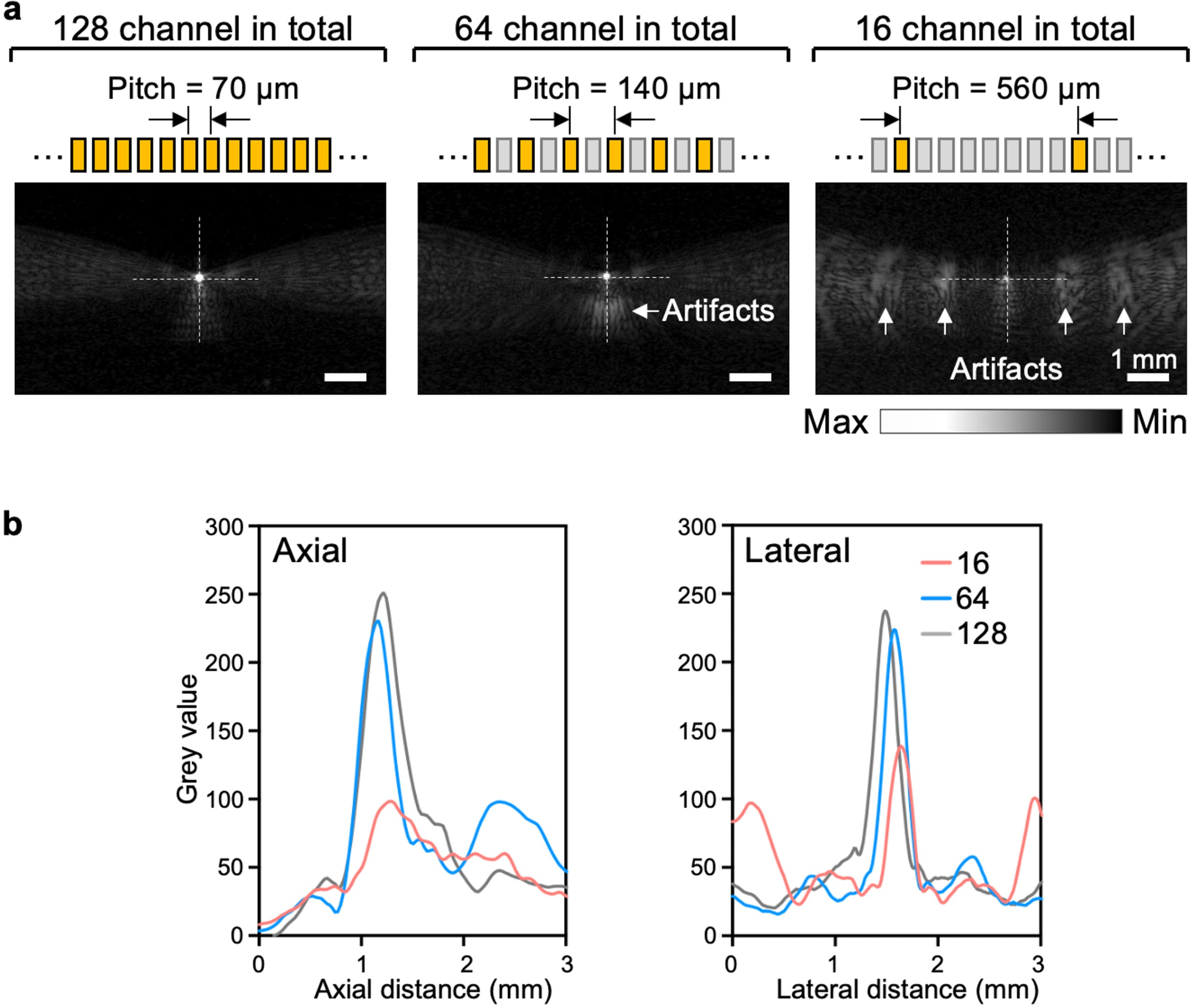

Supplement: S1 Fig — (a) Wire imaging reconstructed from 128-, 64- and 16-channel ultrasound probe. (b) Characterization of axial (left) and lateral (right) resolution of the 128-, 64- and 16-channel probe. (TIF) [file pone.0293468.s001.tif]

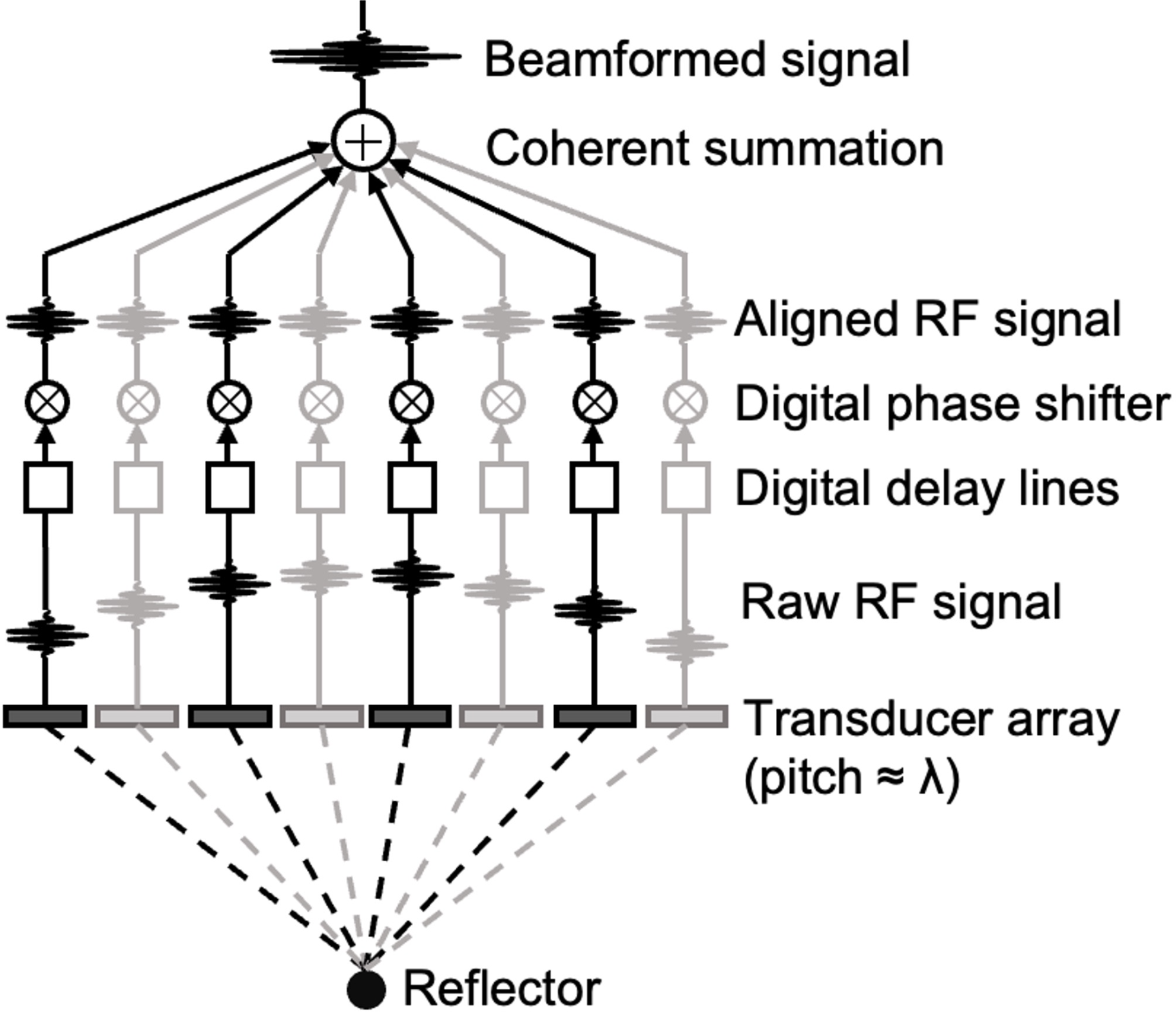

Supplement: S2 Fig — (TIF) [file pone.0293468.s002.tif]

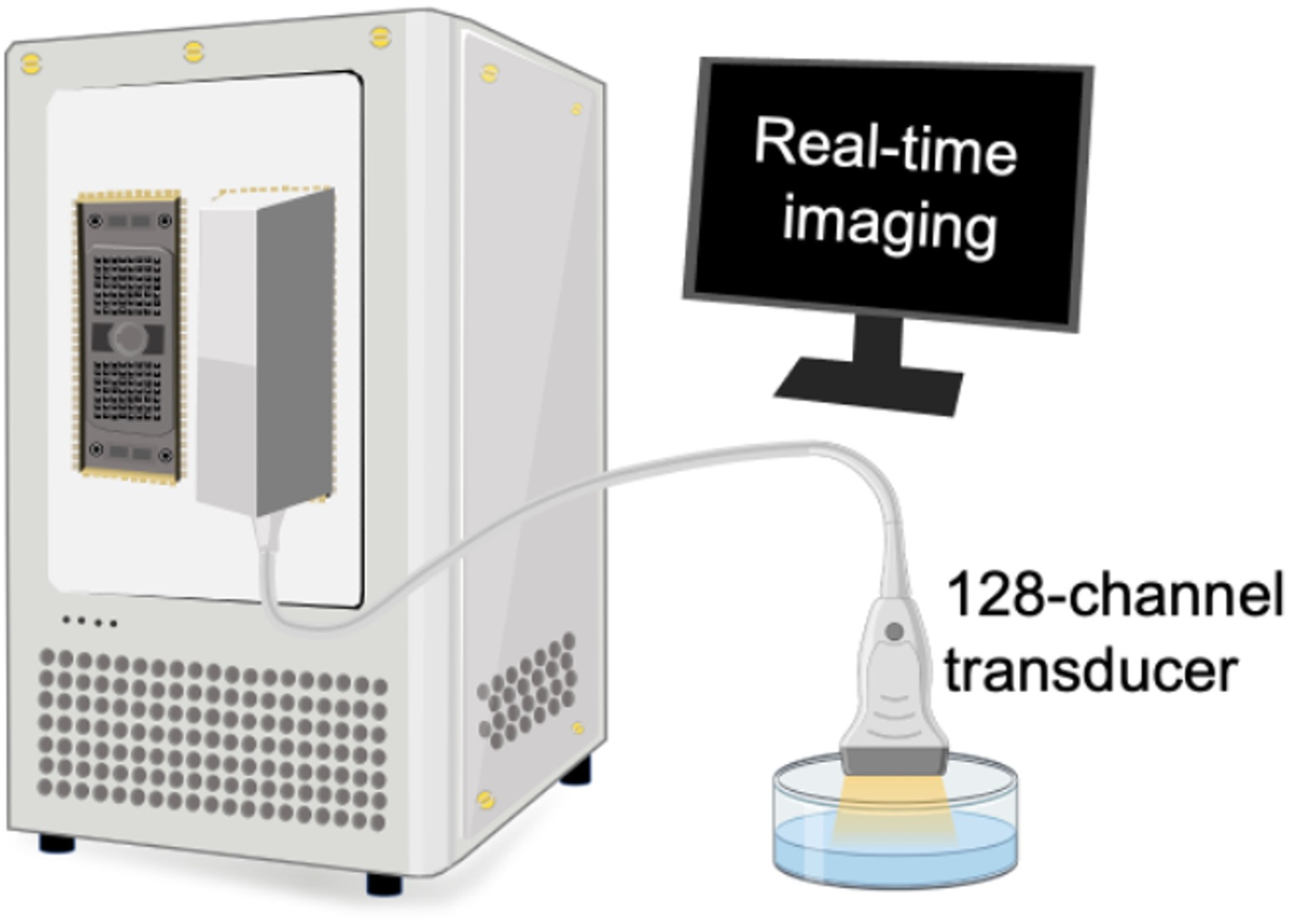

Supplement: S3 Fig — The system includes ultrasound transducer and DAQ system. The ultrasound transducer is connected to DAQ system for power supply, data processing and real-time imaging display. (TIF) [file pone.0293468.s003.tif]

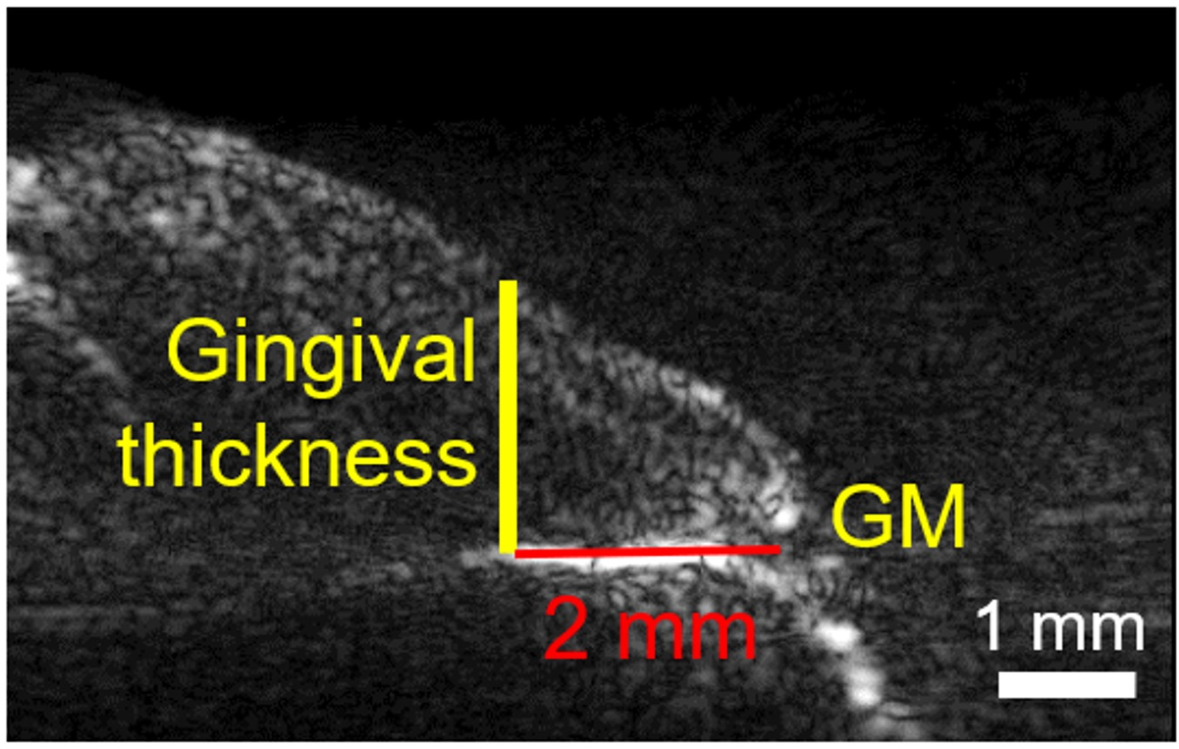

Supplement: S4 Fig — Red line: 2-mm from the GM. Yellow line: gingival thickness. GM: gingival margin. (TIF) [file pone.0293468.s004.tif]

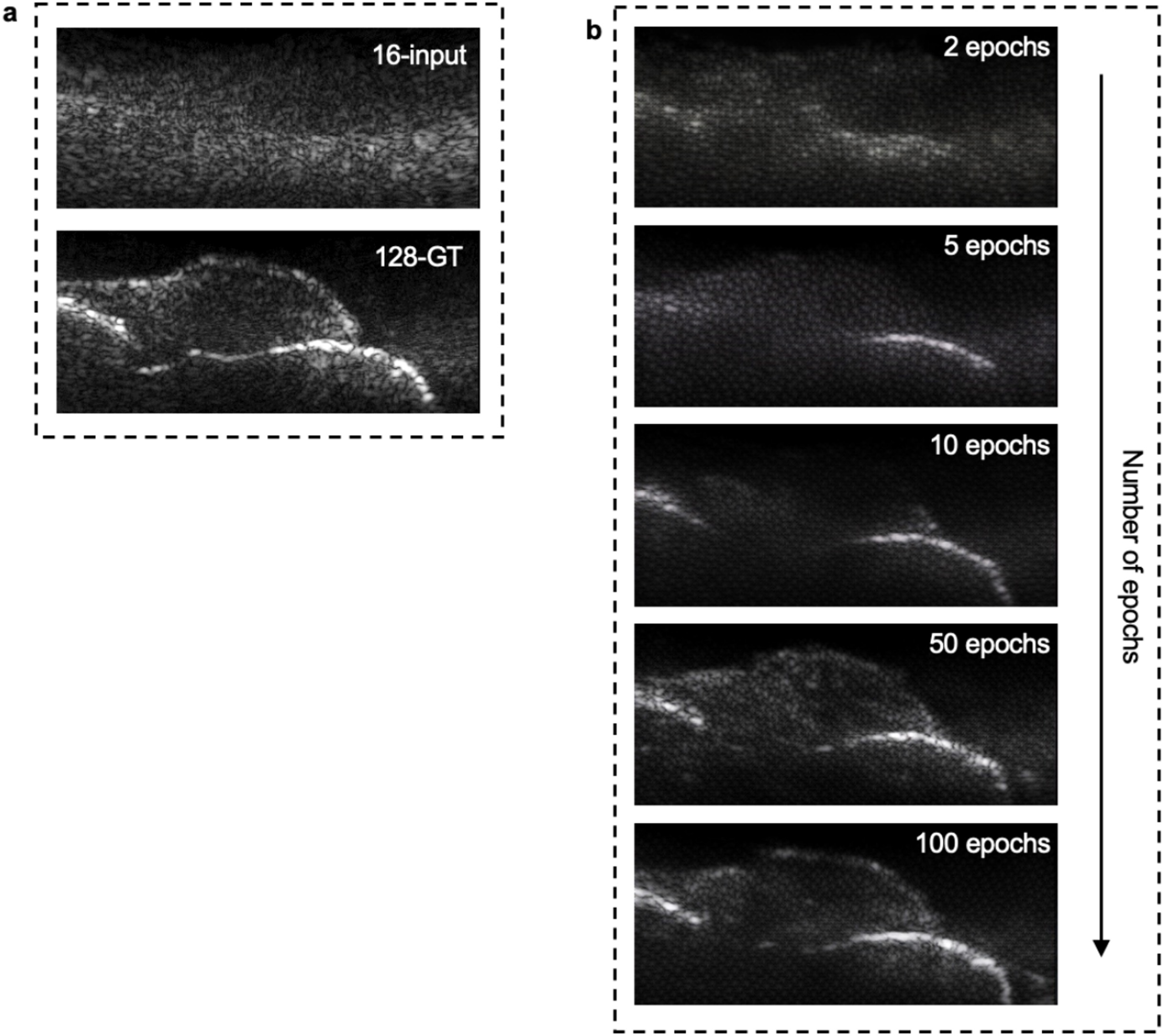

Supplement: S5 Fig — (TIF) [file pone.0293468.s005.tif]

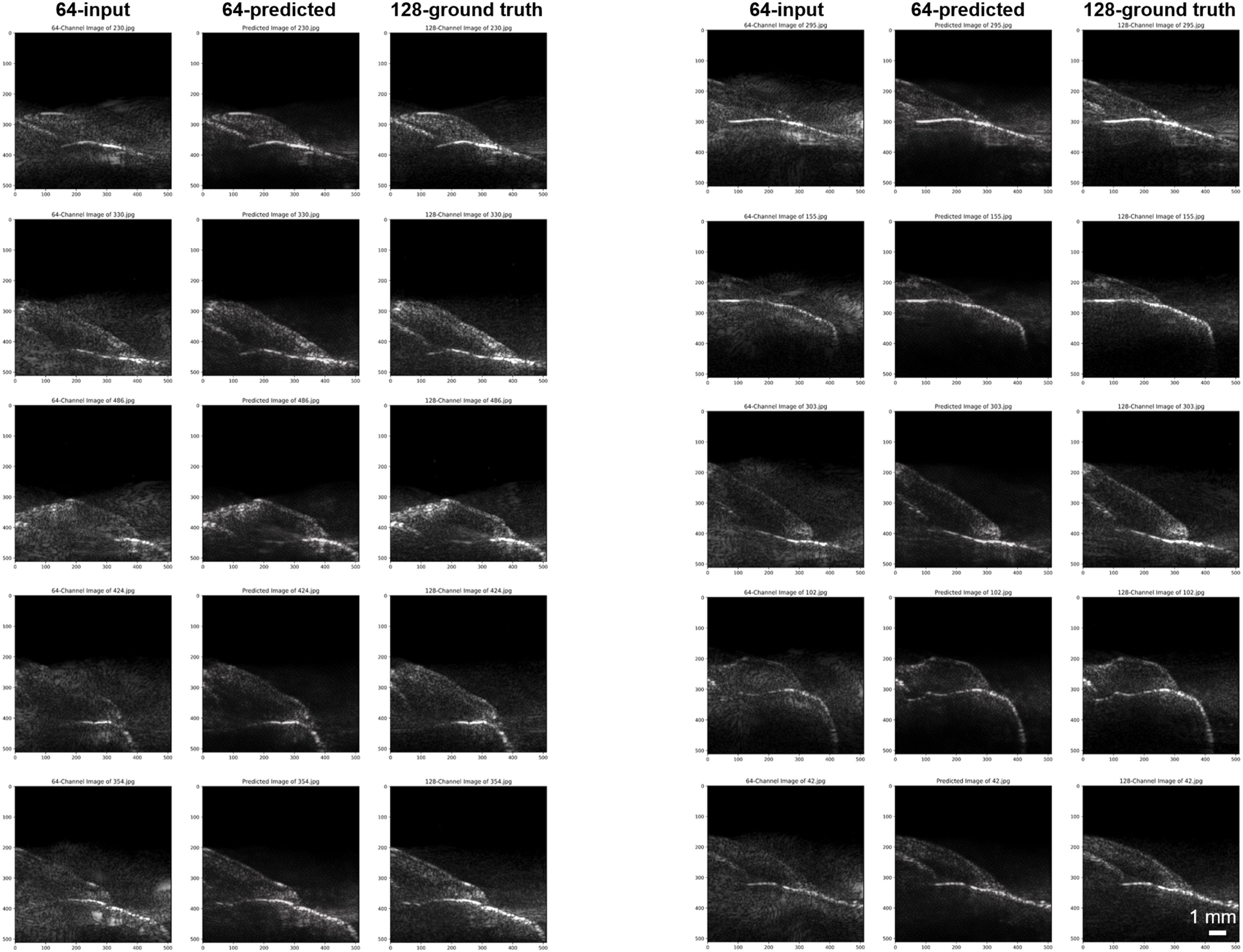

Supplement: S6 Fig — The scale bar applies to all images. (TIF) [file pone.0293468.s006.tif]

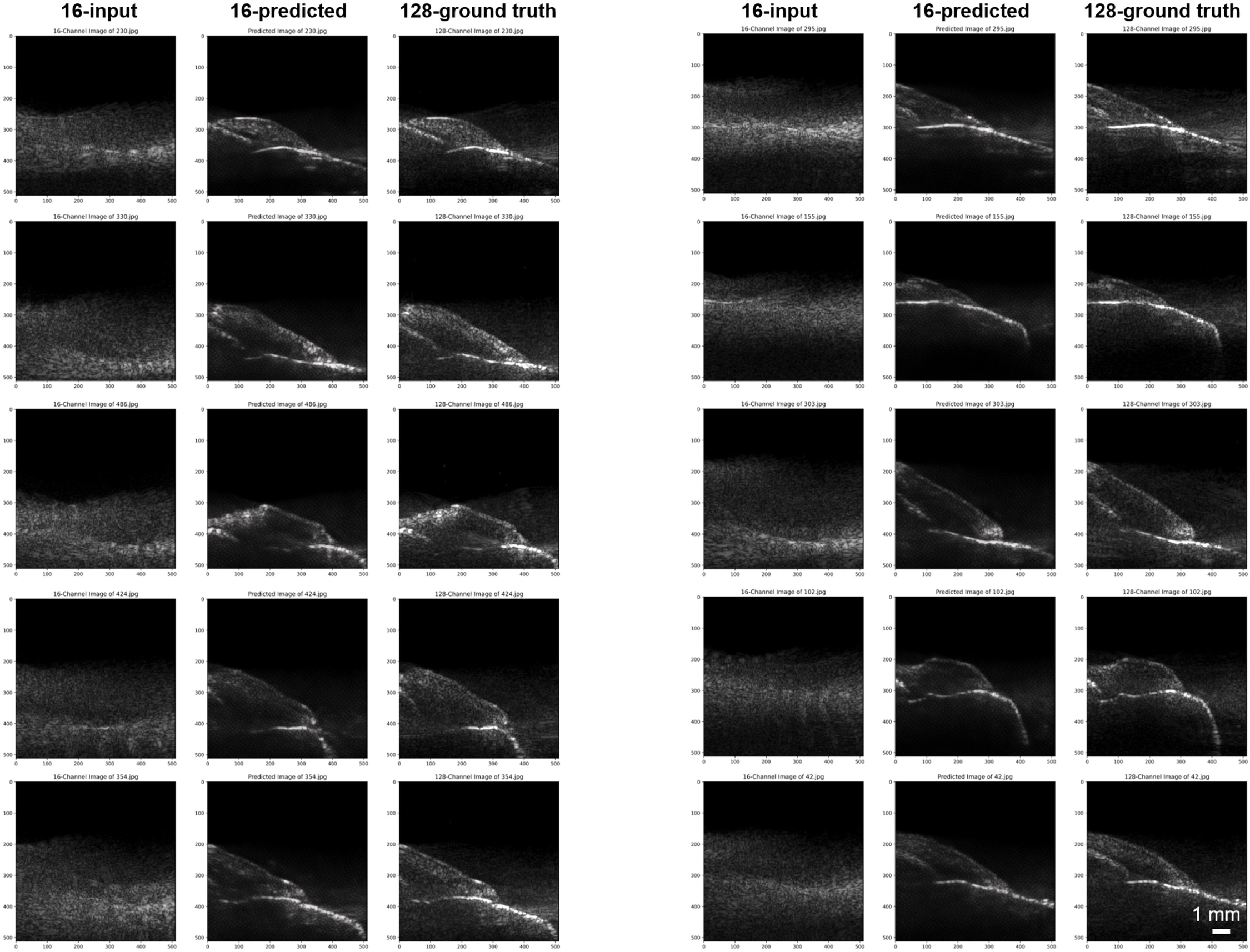

Supplement: S7 Fig — The scale bar applies to all images. (TIF) [file pone.0293468.s007.tif]

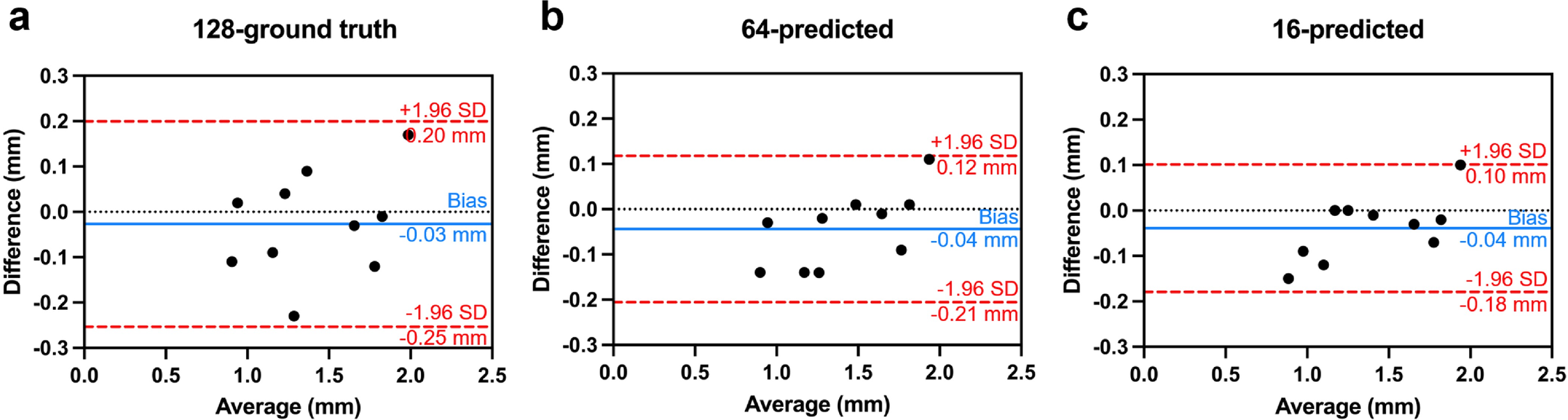

Supplement: S8 Fig — (a) 128-ground truth, (b) 64-predicted, and (c) 16-predicted images. (TIF) [file pone.0293468.s008.tif]

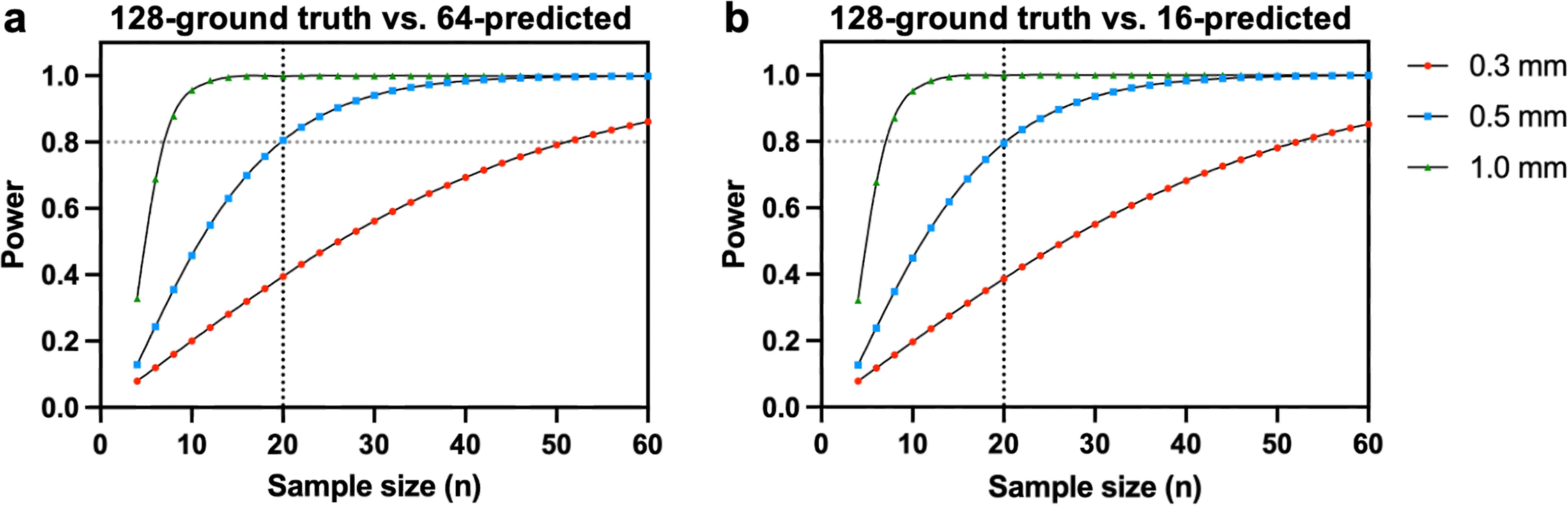

Supplement: S9 Fig — Mean differences were 0.3 mm (red), 0.5 mm (blue), and 1.0 mm (green). Effect size was calculated using gingival thickness measurement standard deviations of (a) 128-ground truth vs. 64-predicted and (b) 128-ground truth vs. 16-predicted, respectively. (TIF) [file pone.0293468.s009.tif]

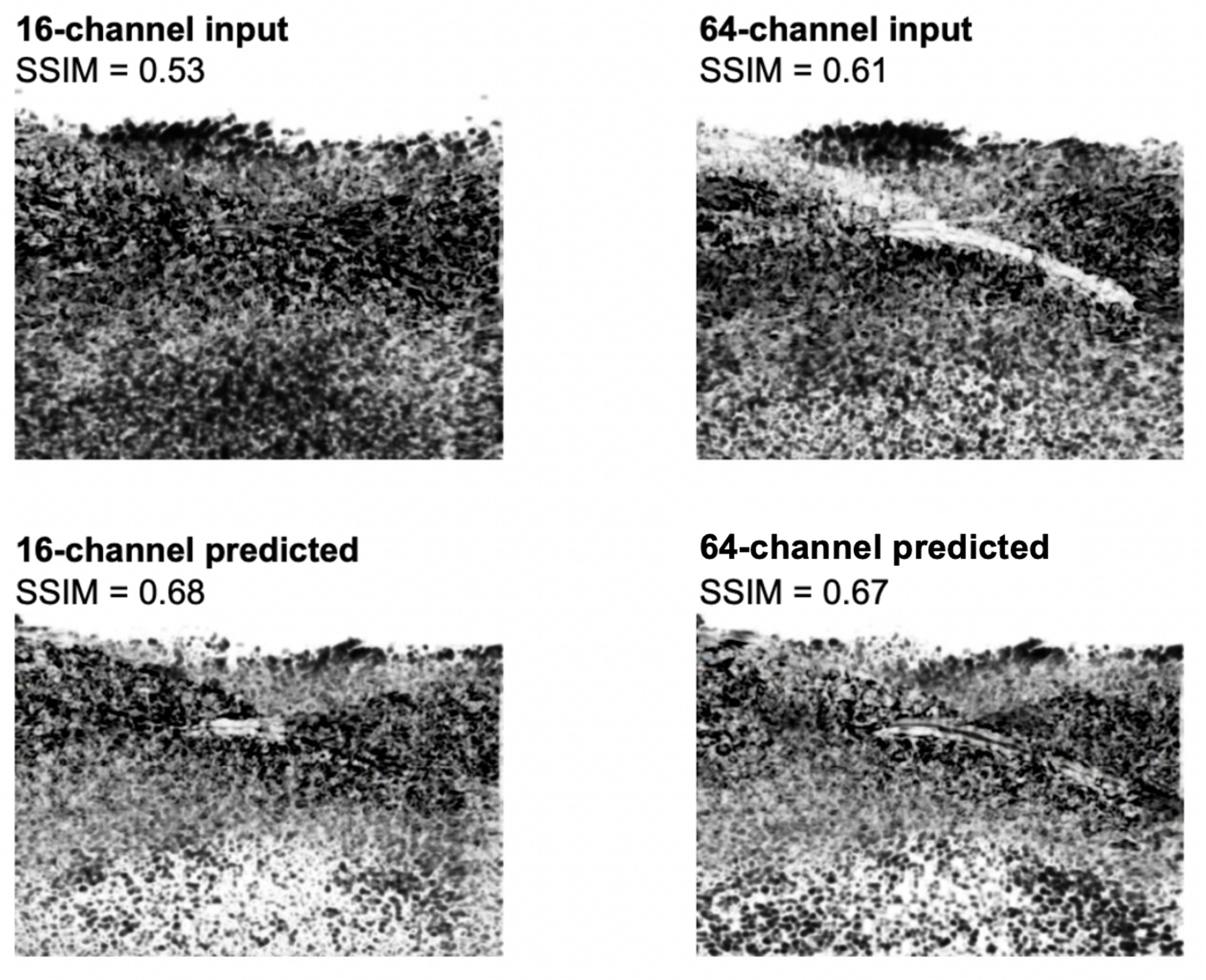

Supplement: S10 Fig — SSIM: structural similarity index measure. (TIF) [file pone.0293468.s010.tif]

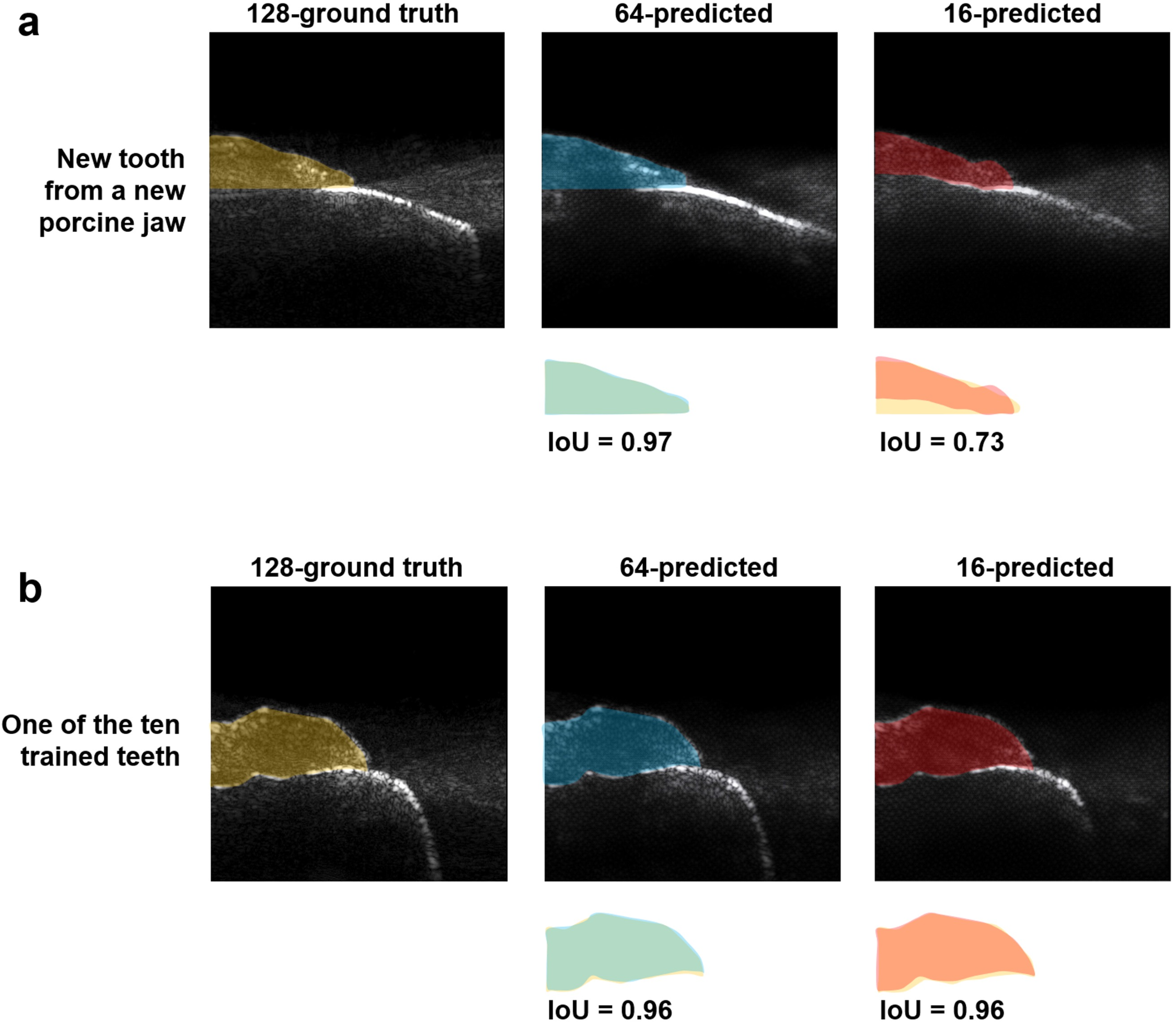

Supplement: S11 Fig — (a) In the new tooth from a new porcine jaw, the IoU of gingiva region was 0.97 for 128-ground truth vs. 64-predicted and 0.73 for 128-ground truth vs. 16-predicted. (b) In one of the ten teeth in the training dataset, the IoU of gingiva region was 0.96 for 128-ground truth vs. 64-predicted and 0.96 for 128-ground truth vs. 16-predicted. Yellow, blue, and red regions indicate the gingiva region of 128-ground truth, 64-predicted, and 16-predicted image, respectively. Green and orange regions indicate the intersection of the 128-ground truth with the 64-predicted and 16-predicted, respectively. IoU: intersection over union. (TIF) [file pone.0293468.s011.tif]

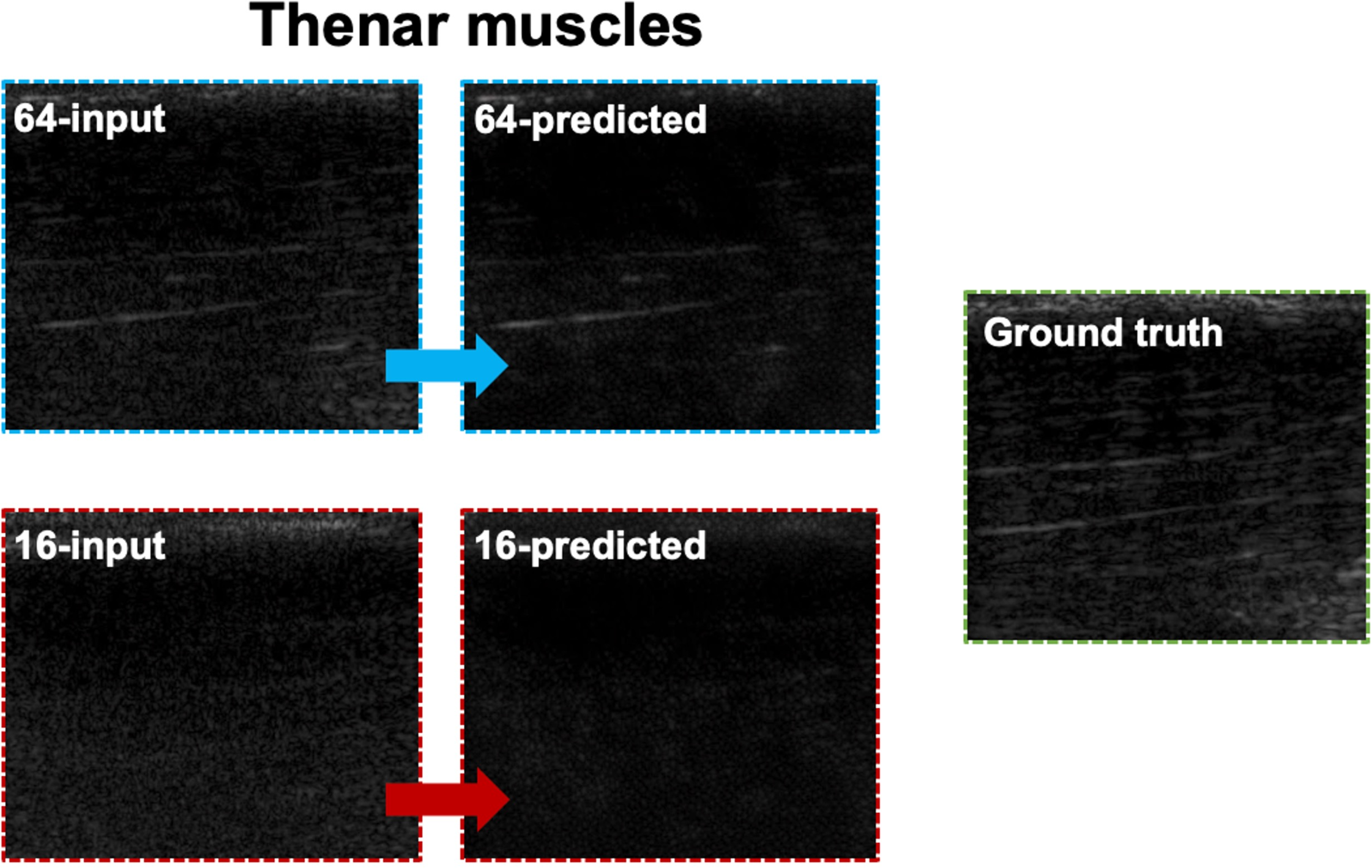

Supplement: S12 Fig — (TIF) [file pone.0293468.s012.tif]
