# Supplementary material for: Deep learning assisted sparse array ultrasound imaging
Source: PLoS One. 2023 Oct 30;18(10):e0293468. doi: 10.1371/journal.pone.0293468 (PMC10615290; doi:10.1371/journal.pone.0293468)
Supplement: S1 Table — (DOCX) [file pone.0293468.s013.docx]

| **Reference** | **Sparse pitch / original pitch** | | **Demonstration** | |
| --- | --- | --- | --- | --- |
|  | **Transmission** | **Receiving** | **Simulation** | **Tissue** |
| [1] | 1/8 | 1 |  | x |
| [2] | 1 | 1/3 | x |  |
| [3] | 7/32 | 1 | x |  |
| [4] | 1 | 1/2 |  | x |
| [5] | 1/2 | 1/2 |  | x |
| **Our work** | 1/8 | 1/8 |  | x |

References

1. Liu Z, Wang J, Ding M, Yuchi M, editors. Deep Learning Ultrasound Computed Tomography with Sparse Transmissions. 2021 IEEE International Ultrasonics Symposium (IUS); 2021: IEEE.

2. Perdios D, Vonlanthen M, Martinez F, Arditi M, Thiran J-P, editors. Single-shot CNN-based ultrasound imaging with sparse linear arrays. 2020 IEEE International Ultrasonics Symposium (IUS); 2020: IEEE.

3. Song J, Liu Y, Ma S, editors. Ultrasonic phased array sparse-TFM imaging based on deep learning and genetic algorithm. 2021 International Conference on Image, Video Processing, and Artificial Intelligence; 2021: SPIE.

4. Kumar V, Lee P-Y, Kim B-H, Fatemi M, Alizad A. Gap-filling method for suppressing grating lobes in ultrasound imaging: Experimental study with deep-learning approach. IEEE Access. 2020;8:76276-86.

5. Xiao D, Pitman WM, Yiu BY, Chee AJ, Alfred C. Minimizing Image Quality Loss After Channel Count Reduction for Plane Wave Ultrasound via Deep Learning Inference. IEEE Transactions on Ultrasonics, Ferroelectrics, and Frequency Control. 2022;69(10):2849-61.
