# Supplementary material for: Deep learning assisted sparse array ultrasound imaging
Source: PLoS One. 2023 Oct 30;18(10):e0293468. doi: 10.1371/journal.pone.0293468 (PMC10615290; doi:10.1371/journal.pone.0293468)
Supplement: S2 Table — (DOCX) [file pone.0293468.s014.docx]

|  | **SSIM** | | **MSE** | | **PSNR** | |
| --- | --- | --- | --- | --- | --- | --- |
|  | **Input** | **Predicted** | **Input** | **Predicted** | **Input** | **Predicted** |
| **64-channel** | 0.66 | 0.73 | 252 | 137 | 24.3 | 27.0 |
| **16-channel** | 0.56 | 0.68 | 511 | 220 | 21.2 | 24.9 |

SD: SSIM: structural similarity index measure.

MSE: mean squared error.

PSNR: peak signal-to-noise ratio.
