# Supplementary material for: Deep learning assisted sparse array ultrasound imaging
Source: PLoS One. 2023 Oct 30;18(10):e0293468. doi: 10.1371/journal.pone.0293468 (PMC10615290; doi:10.1371/journal.pone.0293468)
Supplement: S3 Table — (DOCX) [file pone.0293468.s015.docx]

|  | **Tooth A** | | **Tooth B** | | **Tooth C** | |
| --- | --- | --- | --- | --- | --- | --- |
|  | **Mean (SD)** | **Relative SD** | **Mean (SD)** | **Relative SD** | **Mean (SD)** | **Relative SD** |
| **128-ground truth** | 1.90 (0.03) | 2% | 1.67 (0.01) | 1% | 0.96 (0.01) | 1% |
| **64-predicted** | 1.88 (0.03) | 2% | 1.65 (0.02) | 1% | 0.97 (0.01) | 1% |
| **16-predicted** | 1.89 (0.03) | 2% | 1.67 (0.01) | 1% | 0.96 (0.01) | 1% |

SD: standard deviation.
