# Supplementary material for: Deep learning assisted sparse array ultrasound imaging
Source: PLoS One. 2023 Oct 30;18(10):e0293468. doi: 10.1371/journal.pone.0293468 (PMC10615290; doi:10.1371/journal.pone.0293468)
Supplement: S4 Table — (DOCX) [file pone.0293468.s016.docx]

|  | Tooth A | | Tooth B | | Tooth C | |
| --- | --- | --- | --- | --- | --- | --- |
|  | **Mean (SD)** | **Relative SD** | **Mean (SD)** | **Relative SD** | **Mean (SD)** | **Relative SD** |
| 128-ground truth | 2.07 (0.04) | 2% | 1.64 (0.03) | 2% | 0.85 (0.03) | 4% |
| 64-predicted | 1.99 (0.02) | 1% | 1.64 (0.02) | 1% | 0.83 (0.01) | 1% |
| 16-predicted | 1.99 (0.00) | 0% | 1.64 (0.02) | 1% | 0.81 (0.01) | 1% |

SD: standard deviation.
