# Supplementary material for: Deep learning assisted sparse array ultrasound imaging
Source: PLoS One. 2023 Oct 30;18(10):e0293468. doi: 10.1371/journal.pone.0293468 (PMC10615290; doi:10.1371/journal.pone.0293468)
Supplement: S5 Table — (DOCX) [file pone.0293468.s017.docx]

| **Methods** | **Tooth A** | **Tooth B** | **Tooth C** | **Mean** |
| --- | --- | --- | --- | --- |
| **Clinical probing** | 1.9 | 1.7 | 1.0 | 1.53 |
| **128-ground truth** | 1.99 | 1.66 | 0.91 | 1.52 |
| **64-predicted** | 1.94 | 1.65 | 0.90 | 1.50 |
| **16-predicted** | 1.94 | 1.66 | 0.89 | 1.50 |
